# Musicotherapy mobile applications: what level of evidence and potential role in psychiatric care? A systematic review

**DOI:** 10.3389/fpsyt.2024.1366575

**Published:** 2024-06-07

**Authors:** Gaëtan Leschallier De Lisle, Antoine Oudin, Alexis Bourla, Florian Ferreri, Stephane Mouchabac

**Affiliations:** ^1^Sorbonne Université, ICRIN Psychiatry (Infrastructure of Clinical Research In Neurosciences - Psychiatry), Brain Institute (Institut du Cerveau et de la Moëlle (ICM)), Institut national de la santé et de la recherche médicale (INSERM), Centre national de la recherche scientifique (CNRS), Paris, France; ^2^Department of Psychiatry, Saint-Antoine Hospital, Sorbonne University, Paris, France; ^3^Clariane, Medical Strategy and Innovation Department, Paris, France; ^4^Research Department, NeuroStim Psychiatry Practice, Paris, France

**Keywords:** psychiatry, music therapy, application, mobile phone, mHealth

## Abstract

**Context:**

In our times of smartphone ubiquity, mobile applications are an inescapable daily life tool, including in health care. Music therapy has already proven its worth, notably in mental health. Hence, we were interested in the mobile app format for this type of therapy, its level of evidence, how to use it in daily psychiatric care and the leads for future research and innovation.

**Method:**

This study carries out a systematic review of scientific literature of this topic on two search engines, PubMed and PubPsych, using these key-words: [(web-application) OR (web-app) OR (smartphone) OR (apps) OR (app)) AND ((music) OR (music therapy) OR (melody)].

**Outcome:**

Out of a total of 282 studies found by keyword, 31 are included in this review. Several outcomes emerge. These studies relate to existing applications like Music Care, Calm or Unwind, on application prototypes or a potential use of music streaming applications on health care. They involve many different populations and clinical situations, including in hospital environments, for patients with chronic illnesses, different age ranges or for the general population. These musical interventions show a significant effect mainly for anxious symptoms, but also for depression, sleep disorders, pain and other psychiatric or psycho-somatic syndromes. These applications have no significant adverse effects.

**Conclusion:**

This review shows that music therapy apps have several potentials for improving mental health care. It could assist psychiatric usual care and could potentially lower medication intake. Nevertheless, the studies on the topic are limited and recent but they open prospects for future research.

## 1 Introduction

Music therapy, as a non-pharmacological therapeutic approach, has gained recognition and popularity over the decades for its potential to improve mental health and well-being. Music has been recognized, second only to sports, as the most effective activity for enhancing mood, increasing alertness, and promoting relaxation (1). Music therapy uses the power of music as a means of communication, expression, and treatment of psychiatric disorders, with a well-documented effectiveness in major psychiatric pathologies. Indeed, a recent meta-analysis (2) describes its utilization in the treatment of the five major psychiatric disorders: schizophrenia, bipolar disorder, generalized anxiety disorder (GAD), major depressive episode (MDE), and post-traumatic stress disorder (PTSD). The studies primarily consist of randomized clinical trials, which potentially indicate quality, although the authors advise caution, and the outcomes encompass various forms of treatment for these illnesses, including symptom improvement, affective changes, quality of life, functional, social, or emotional impact, access to care, etc.

While still relatively unknown, the musical brain has already been the subject of extensive research. The musical brain can be summarized with the temporal lobe, particularly its upper part, where the auditory cortex processes the fundamental elements of music, such as notes, rhythms, or melodies. On the other hand, the frontal lobe analyzes the more complex structure of music, its phrasing, and links it to motor functions. The pleasurable and motivational experience provided by music is directly linked to the activation of the dopaminergic system (3, 4).

Mental health is a global concern with an increasing prevalence of psychiatric disorders such as depression, anxiety, schizophrenia, and bipolar disorder (2). Despite advancements in psychopharmacology and psychotherapy, many patients remain undertreated or lack adequate access to therapeutic interventions. In the current context of the fourth industrial revolution (5) and the rise of smartphones, mobile health is gaining importance and offering new therapeutic avenues through what is called “mHealth” with mental health being the leading category in terms of the number of applications, according to IQVIA (6). In this field, digital music therapy applications are emerging as a promising alternative, providing easy access to personalized musical interventions and potentially effective solutions for improving mental health, but what is the efficacy of mobile music therapy applications in the treatment of psychiatric disorders?

This article proposes the first review of the scientific literature on the overall offering of mobile psychiatric care applications through music. It aims to provide a user guide for both individuals and professionals, assessing the benefits and limitations of these applications in the management of psychiatric disorders and symptoms while highlighting areas that require future research.

## 2 Methods

### 2.1 Selection criteria

#### 2.1.1 Inclusion criteria

As this article aims to explore the literature at the intersection of psychiatry, mobile applications, and music therapy, there are no specific temporal or locational criteria. Below are the inclusion criteria for the review:

##### 2.1.1.1 Population

All patients diagnosed with a psychiatric disorder who are capable of participating in a music-based intervention, without age, gender, care setting, or residential location restrictions. Patients with non-psychiatric disorders where the psychological component is examined, such as pain or severe illness, may also be included. Additionally, any population experiencing stress, mood fluctuations, or sleep disturbances, where these parameters are related to the study’s objective, will also be included.

##### 2.1.1.2 Intervention

Any form of music intervention or therapeutic use of music, whether active or passive, individual or group-based, as long as it is mediated or directly involves a mobile application or web application and aims to treat or alleviate symptoms related to psychiatric disorders or psychological aspects of non-psychiatric conditions. The application must be the therapeutic tool used in the music intervention; studies using an application solely as a measurement tool will not be included.

##### 2.1.1.3 Study design

All study designs are eligible, including randomized controlled trials, qualitative studies, case reports, systematic reviews, meta-analyses, etc. Any form of control, whether standard treatment, placebo, or another treatment under investigation, will be included.

##### 2.1.1.4 Outcome

Any results that demonstrate or support the utility of music therapy applications in mental health care, including changes, reductions, or total resolution of symptoms; changes in quality of life, functional, emotional, or social impact; access to care, or other relevant outcomes, will be considered.

#### 2.1.2 Exclusion criteria

All publications with irrelevant topics or those appearing unrelated to considerations such as the impact on care organization, ethical implications, or potential applications of digital technology within the field.

### 2.2 Search criteria

To adhere to international standards for a systematic literature review, two databases were consulted: PubMed and PubPsych. The following keywords were used for the review: [(web-application) OR (web-app) OR (smartphone) OR (apps) OR (app)] AND [(music) OR (music therapy) OR (melody)].

For the search strategy, we read all the articles found in the databases with the keyword, no other filters nor limits were used. For the study selection, only one reviewer included the studies according to the inclusion criteria. He worked independently and no automation tool was used in the process.

Concerning the data collection process, one reviewer collected data from each report, he worked independently and no automation tool was used in this process. For the assessment of bias, all the co-author participated, worked independently and no automation tool was used.

## 3 Results

**Figure 1:**
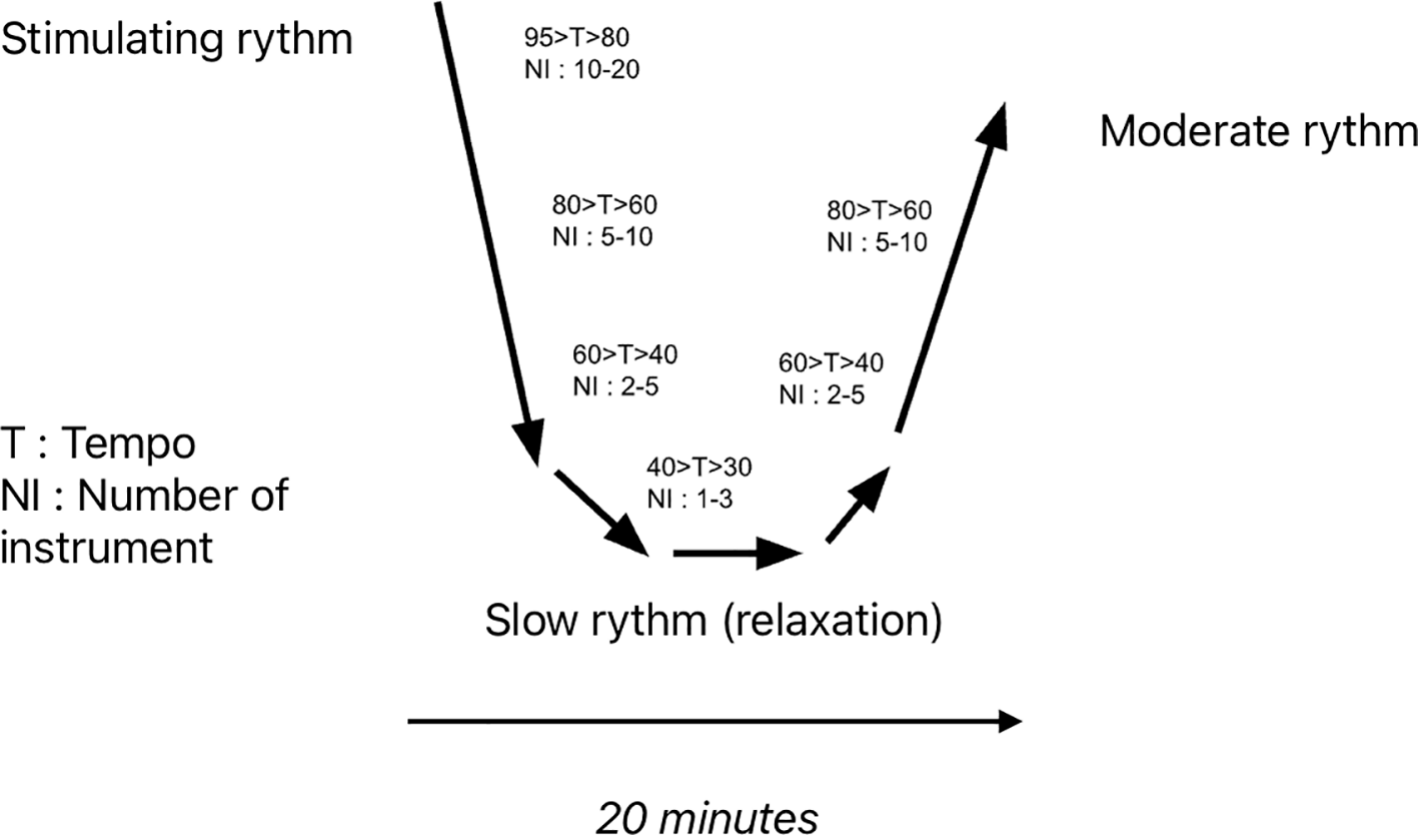
Diagram depicting a U sequence.

With the above keywords, I was able to find 229 results on PubMed and 53 results on PubPsych. The vast majority (250) were off-topic, and one study was a duplicate of another, leaving me with a final inclusion of 31 studies in the review (**Figure 1**):

The studies focus on three specific healthcare contexts: everyday life and the general population, chronic diseases, and hospital settings.

### 3.1 Everyday life, general population

These studies focus on psychiatric symptoms encountered in the general population, particularly anxiety and sleep disorders. While some investigations specifically target applications such as Music Care or Calm, others conduct literature reviews on the use of mental health applications. Some studies compare music therapy to other therapies mediated by mobile applications, while at last others adopt a more conceptual approach.

#### 3.1.1 Application specific studies

Some applications aimed at the general public have already been scientifically evaluated, such as the Calm application, which has a significant effect on sleep, especially for falling asleep (p < 0.001), as well as for anxiety (p < 0.001), depression (p < 0.001), and post-traumatic stress (p = 0.026) (7). Calm application has been studied (8) by surveying parents about their children’s use of the Calm application. Out of 3000 surveyed adults with children, half have children using the application. Among the features offered by the application, the Music/Sound Environment tool is the second most used, with 67% utilization. 78% of parents describe this tool as useful. The application is primarily used for sleep disorders, with playlists of 3 to 4 hours that you can set to stop with a timer.

**Figure 2:**
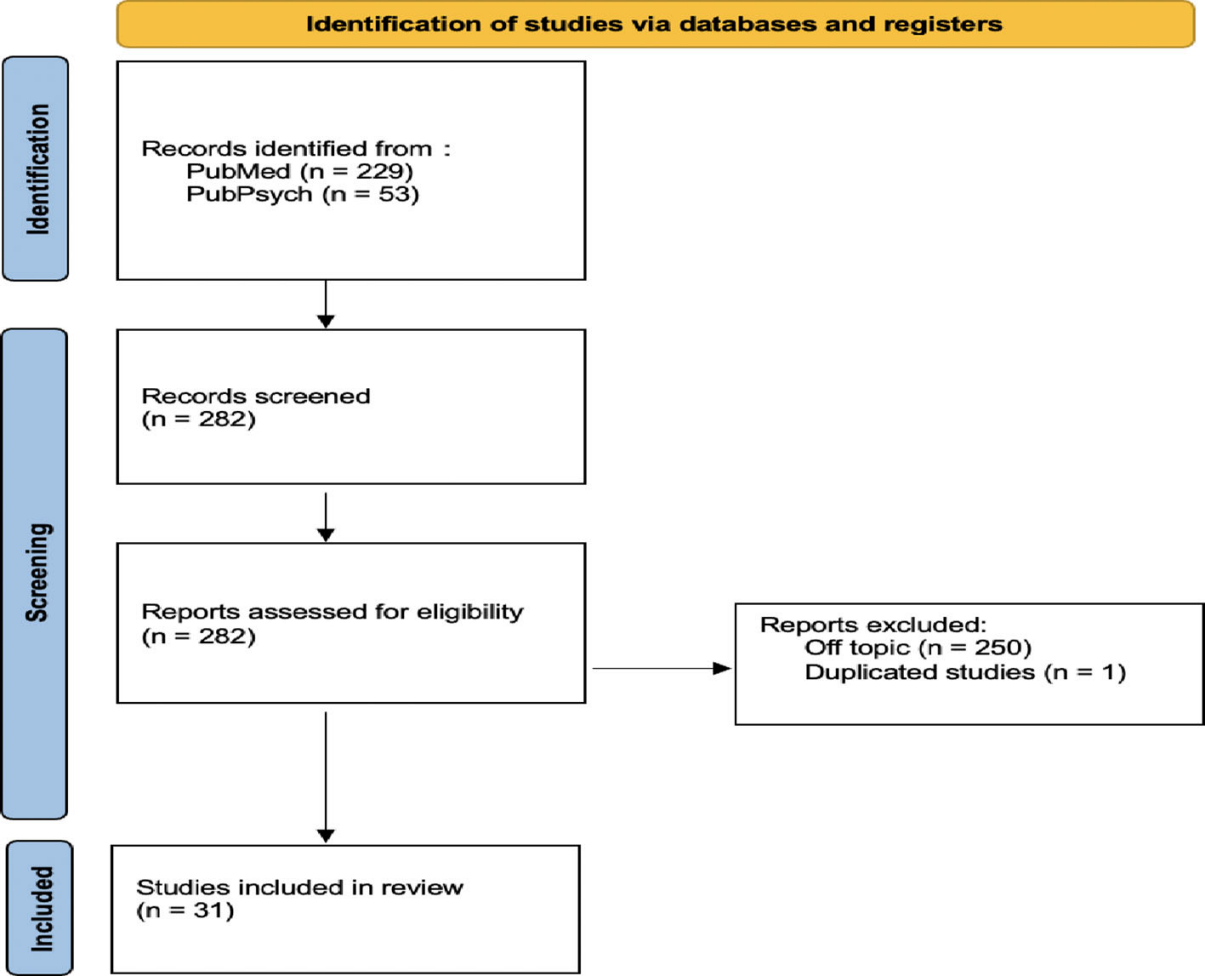
PRISMA Diagram of the systematic review in this article, according to the PRISMA recommendation statement (10).

The Music Care application has also been evaluated for these indications in the general population, like for symptoms of stress and sleep disorders in professional football coaches, with significant effectiveness (9). This app uses specific music sequences, the most used being the U sequence (**Figure 2**). The U-shaped method involves a sequence of musical pieces, smoothly faded and linked together, divided into several phases, with a gradual reduction followed by an increase in tempo, the number of instruments, and volume. This method aims to achieve a psycho-physiological effect on the patient (e.g., Sleep induction: gradual reduction of musical components; Awakening: progressive increase of musical components). The patient lies down and listens to the sequence through headphones. The musical pieces are selected based on the patient’s preferences, with a wide range of styles available (classical, contemporary, jazz, popular, rock, electronic, world music, etc.). Typically, the sequence lasts for 20 minutes. They also use L or J sequences, respectively aimed at promoting sleep or arousal, corresponding to the first half of the U sequence or the second half, spanning on a period of 20 minutes.

#### 3.1.2 Multiples apps review

Review studies of mobile applications in mental health care show the growing role of music therapy in this expanding field. For example, Blazquez et al. (11) conducted a review of m-Health in the field of stress management by evaluating applications available on the market. Out of the 443 applications included in the study, the most prominent category (22%) is that of musical intervention, referred to as “relaxing music.” They mention Calm application, as well as Qi Gong Meditation and Relax Lite as noteworthy applications offering music therapy.

Schlarb et al. (12) conducted a review of applications for childhood sleep disorders. They identified 573 applications on Google Play and the Apple Store, with 39.4% using “lullabies,” 35.9% (206) using “sleep-oriented music,” and 7.7% (44) using songs. Musical intervention is thus the predominant approach. Among all these applications, 19 have been installed more than five million times and they primarily use music or sounds.

A review by Hwang et al. (13) specifically dedicated to mental health applications for adults screened 7 search engines, but only fourteen studies met their criteria. Two applications are explicitly described as using musical intervention and demonstrating significant results: “It’s time to relax” and “Mind Healer.”

#### 3.1.3 Comparison to other therapies - conceptual articles

Music therapy is also studied in comparison to other therapies that also use mobile applications. Whether compared to a Mindfulness application (14, 15) or language- mediated psychotherapies in general (16), music therapy applications show no significant differences from other interventions that have already proven their effectiveness and demonstrate significant effectiveness on stress symptoms, especially those related to pain and physical Problems (p = 0.026) (14), and if the intervention is closely aligned with the stressful moment, in an acute context (15, 17).

To understand this effectiveness, Clarke et al. (18) propose to gather all available data on the relationship between music, empathy, and openness to other cultures. They describe musical empathy as a sensitivity that combines innate and acquired aspects, linked to mirror neurons, capable of deeply changing our behavior and, in the context of the study, of opening us up to others, to different cultures, and to different emotions. This musical empathy is described as entirely applicable to passive music listening and the format of music therapy applications.

Taruffi et al. (19), on the other hand, delve into the concept of mind wandering, with its connection between everyday music listening, mood, and the content of thoughts. Mind wandering is defined as a shift away from the external world in favor of an internal awareness directed toward a stream of thoughts or images, representing up to 50% of a human being’s waking time. this prevalence increases when listening to music. Subjects report an improvement in mood and wakefulness after listening to the offered music, describing a sense of peace (most frequently reported), nostalgia, tenderness, and wonder. Positive thoughts are reported more frequently and intensely, with a significant link between the two, indicating that emotions induced by music directly influence the content of thoughts.

Summary table in the **Appendix**.

### 3.2 Chronic conditions

Whether for patients with dementia, epilepsy, sickle cell disease, cancer, migraine, or severe psychiatric disorders, these studies are equally concerned with the effectiveness of existing music therapy applications and the future of digital therapies in the field.

Concerning patients with dementia, Tak et al. (20) conducted a review of 83 applications used to improve symptoms and the quality of life for these patients. These applications aim not only to enhance cognitive abilities but also to address psychiatric symptoms such as apathy, irritability, agitation, social isolation, or sleep disturbances. More than 10% of these applications specifically involve music, either through simple listening or active music engagement (**Table 1**). The authors emphasize the personalized aspect of music applications, their ability to adapt to the patient’s preferences, the meaningfulness of the application, interactivity, and user-friendliness.

**Table 1:** Musical interventions reported by the study of Tak et al. (20).

Music, sound, and musical instruments	Use fingers to hit virtual simulated musical instruments to play on the screen and enjoy music without having to know playing techniques and musical knowledge	Simulated musical instruments (e.g., piano, congas, harp, percussive, Tongue Drum, Cajon), Virtuoso
	Create and record and share relaxing and pleasant melodies by tapping the screen along with their accompanying simple but colorful visualizations, a simple composition tool without musical knowledge and experience (e.g., Bloom) Choose and listen to personalized radio stations (e.g., Pandora)	Bloom, Raindrops, Healing Voice Lite
	Uses ambient sounds, nature sounds, realistic wind chimes, pleasant melodies, and binaural beats with a visual escape for relaxation and meditation	Just Chimes, Relaxing sounds of nature
	Listen to old time radio shows in the 1920s-1950s	Yesterday USA, Pandora

For the same dementia patients, Zhang and Ho (21) propose a work on reminiscence therapy by developing a mobile application using open-source data, i.e., pre-existing, refined code made available to the public for rapid application development. They then test such an application, “S3 music therapy,” designed in this manner over 5 days, for “non-cognitive” symptoms, specifically psychiatric symptoms associated with these conditions.

In the case of epileptic patients, Afra et al. (22) described in 2018 the development of a mobile application with musical intervention to address the depression and anxiety to which these patients are particularly prone. With a 10-minute evening listening format, significant effectiveness is observed, possibly attributed to the activation of the parasympathetic nervous system, which is associated with calm and relaxation. They also mention two applications, SuperBetter and Moodhacker, which significantly reduce depressive symptoms through musical intervention.

For these same epileptic patients, Metcalf et al. (23) attempt to evaluate the analgesic and anti- epileptic effects of music using mouse models and explore the potential use of music through applications, considering the concept of a medication-device duality. The efficacy of musical intervention in pain reduction is tested by comparing it to other analgesic substances, showing significantly lower pain, inflammation, and seizure frequency in the music-treated group (respectively p < 0.05, p < 0.01, and p < 0.05), without differences compared to the drug-treated groups for pain and inflammation. The authors suggest that music’s effects also extend to the sleep phase, indicating an influence on the brainstem and midbrain in addition to the forebrain. The authors describe these effects as more pronounced when the music is chosen by the patients themselves, with potential applications to various chronic diseases, particularly in the field of psychiatry.

For sickle cell patients, Rodgers-Melnick et al. (24) tested a music therapy protocol using mobile applications, resulting in significant improvements in pain management ability (p = 0.016), self- management skills, sleep disturbances (PROMIS scale, average -1.49 ± 6.68, p = 0.023, d = −0.99), and social functioning (ASCQ-Me scale 2.97 ± 6.91, p = 0.018, d = 1.05). Patients met with the therapist once a week for six weeks, with exercises to be performed daily at home as prescribed by the therapist. Several applications (Relax HD, GarageBand PianoScale, or music streaming applications) were tested for a wide range of exercises.

For cancer patients and survivors, Huberty et al. (25) developed a meditation application prototype inspired by the Calm application. This application offers various options, including a Music feature. Based on their experience with Calm or personal preferences, the subjects explicitly request that the application include musical intervention, expressing that this feature would benefit both the cancer- affected population and those caring for cancer patients, as well as the general population using the application.

For the treatment of episodic migraines, Parlongue et al. (26) tested the efficacy of the Music Care application in 2021, involving a 20-minute intervention with a U sequence chosen by the patient, tested on 20 patients over three months. The results not only demonstrate a reduction in migraine frequency (the primary outcome measure) but also a significant decrease in medication use, duration of migraines, and anxiety and depressive symptoms.

For patients experiencing pain, Chai et al. published a study in 2017 (27) on the theoretical rationale and appeal of music therapy applications as adjuncts to opioids. They highlight the numerous and obvious problems associated with opioid use, especially in the USA, where the opioid epidemic is still ongoing. They also discuss the time and financial constraints of cognitive-behavioral therapy (CBT) and “intervention fatigue.” They describe the neurological action of music and its link to pain, particularly through the increase in opioid receptors and its effect on the anxiety dimension of pain. Regarding music selection, they emphasize automatic choices. They argue that an artificial intelligence algorithm can continually offer original choices while aligning with the patient’s musical preferences.

For patients with chronic psychiatric conditions, Noel et al. (28) examine the use of electronic devices, particularly applications, in the context of recovery. Recovery in psychiatry encompasses healing, self-management of illness, progress toward personal goals, and a sense of responsibility. Listening to music is the most reported behavior to facilitate this recovery (60%). These patients also express a significant interest in learning how to use psychiatric applications.

For the same psychiatric patients, Schriewer and Bulaj (29) published in 2016 an article on the use of music streaming services for treatment and prevention. They describe a connection between electrodermal activity (EDA), EEG parameters, and the effect of music on the psyche. With connected devices such as watches, it becomes possible to measure these parameters and provide personalized music choices through a biofeedback system, in addition to direct user choices. The authors suggest that daily music listening may have effects on depression (stimulating and positively valenced music), anxiety (calm and soothing music), maintaining euthymia in patients with bipolar disorder (30 minutes of a balance between calm and stimulating music), as well as other psychiatric conditions, pain, and preventive measures.

Finally, for chronic diseases in general, Bulaj et al. (30) investigate the use of digital technologies for self-care. They include music in self-care practices (**Table 2**), along with physical activity, meditation, and quality sleep, as complementary to medical and/or pharmaceutical treatment. They mention several applications in their appendix, for which studies have explored their effectiveness (**Table 3**).

**Table 2:** Effects of self-treatment with music and the physiological explanation of these effects.

Listening to music	Reduction of depression levels (31, 32)	Modulation of parasympathetic nervous system Mojtabavi et al. (33)
	Lowering BP, decrease of anxiety and depressive symptoms in breast cancer patients Wang et al. (34)	Modulation of the immune system Chanda and Levitin (35) C Reduction of cancer-related fatigue Qiu et al. (36)
	Reduction of cancer-related fatigue Qiu et al. (37)	Modulation of opioid receptors Mallik et al. (38)
	Reduction of pain Garza-Villarreal et al. (39); Lee (40)	Modulation of opioid receptors Mallik et al. (38)
	Reduction of epileptic seizures Yuen and Sander (41); Rafiee et al. (42)	

Table adapted from the study by Bulaj et al. (30).

**Table 3:** Applications offering musical interventions. Table adapted from the study by Bulaj et al. (30).

Music	*Memory Tracks*	Mobile app: symptom reduction in dementia patients.
	*Music eScape*	Mobile app: emotion regulation in young people.
	*MusicGlove*	Video game: music-based rehab for stroke patients.
	*Unwind*	Mobile app: patients in ED with acute pain.

### 3.3 Use in hospital settings

In the hospital context, the effectiveness of music therapy on psychiatric symptoms becomes intricately linked to the management of pain and the associated anxiety. One application in particular stands out, Music Care (43–45) with its U, L or J sequences.

Whether in an emergency department (46), for the discomfort of non-invasive ventilation (43), pre- coronary angiography (Guétin et al. (45)), in a surgical department during all perioperative phases (44, 47–49), and Guerrier et al. ), a musical intervention in the form of an application demonstrates a significant impact on pain, anxiety, and quality of life, whether as a standalone intervention or integrated into a nursing care application (49), or even more broadly in an enriched environment (48).

For more specialized services, such as geropsychiatry, Berge et al. (50) described in 2022 the prototype of the Alight application, which includes a musical intervention designed for the “dementia patient” and their “caregiver” dyad. The prototype of the application offers a “digital session” lasting 15–20 minutes, consisting of personalized music accompanied by custom-added images or films, aimed at stimulating musical memory. This intervention aims to improve not only mood but also behavioral symptoms, with predominantly positive results.

In pediatric oncology, a systematic literature review by Frasier et al. (51) on “tablet therapy” underscores the value of distraction in managing pain, to “break the cycle of anxiety and distress” and reduce “threat bias.” Musical intervention is often incorporated into a multifunctional tool or application, which is another advantage of these applications. However, a few applications are specifically dedicated to music therapy, such as Six Strings, Doodle Sounds, or Songza.

## 4 Discussion

This review reveals the diverse applications of music therapy in addressing psychiatric symptoms. Significant improvements are reported for symptoms such as anxiety, mood-related sadness, insomnia, as well as social functioning, mood fluctuations, discomfort, hypertensive events, and pain. The effectiveness is observed among challenging patients who may be resistant to other approaches, such as those with high levels of catastrophic thinking in anxiety. On average, the studies suggest interventions lasting 15 to 20 minutes, a duration found to strike a balance between effectiveness and real-world or hospital applicability (2, 7, 9, 11).

Effectiveness appears to be more pronounced when the intervention occurs close to the targeted symptom, such as during moments of anxiety or just prior to it in acute care or surgical procedures, or in any anxiety-inducing situation. For chronic symptoms, a daily session at a fixed time is generally recommended (14, 17, 25, 46). The most frequently mentioned applications are Music Care and Calm. However, it’s worth noting that, as of now, none of these applications are reimbursed.

In arguments supporting the validity and value of music therapy applications, the universality of music across cultures is emphasized, extending the scope of music therapy to all patient types.

Various populations are studied, including those from the general population, individuals with chronic conditions, and those in hospital settings. Music therapy applications have the advantage of not requiring prior training and are therefore applicable to populations with altered or developing cognitive abilities, such as children (12, 18, 19, 29).

Many studies also cite economic advantages. Mobile applications, without replacing the role and contribution of a human therapist, offer a cost-effective and accessible alternative for a broad range of individuals, especially for disadvantaged populations (6, 20, 30, 43).

Music therapy is also advantageous in its ability to be customized to the patient’s musical preferences, desires, and needs. As applications provide access to an infinite music selection via the internet and streaming platforms, the music recommendation algorithms are powerful enough to be potentially therapeutic. These algorithms can consider various parameters, both intrinsic (e.g., heart rate, electrodermal resistance, ECG and EEG data, regular self-assessments) and extrinsic (e.g., season, weather, temperature, etc.), all of which can influence the choice of music that would be ideal for the patient at a given moment. These algorithms can also benefit from regular updates, easily applicable to all downloaded applications simultaneously (3, 4, 23, 27).

The possibility of continuously refreshing content gives these applications an advantage over other forms of mental health treatment in the form of applications, such as guided meditations, breathing exercises, message-based psychotherapies, which may induce “intervention fatigue” over time, especially for chronic conditions requiring years of treatment (15, 16, 22, 26).

The majority of experimental studies in this review consist of acceptability or feasibility studies with small sample sizes. This prevalence demonstrates the emerging but evident interest in the subject within the research community and provides numerous avenues for future research. One potential extension could involve investigating the efficacy of music therapy applications for acute psychiatric symptoms or physical symptoms exacerbated by anxiety (21, 24, 28, 44, 45).

These studies primarily serve to demonstrate the effectiveness of music therapy in the form of applications and validate the application format in healthcare, regardless of the therapeutic method used. The distraction effect described for music could resemble a dissociative effect and potentially enhance the efficacy of dissociative treatments such as ketamine, or even experimental treatments like psilocybin or LSD, particularly given the suggestive impact of music that may be augmented by these treatments (8, 47–51).

It should be noted that interventions are primarily tested with individual audio devices such as headphones or earphones. Therefore, the effects may be less validated for ambient listening through speakers or live performances, which provides yet another avenue for future research (1, 50).

## 5 Limitations

While music therapy applications may demonstrate their efficacy in these studies, they also have their limitations. To begin with, the number of studies is relatively small, and the subject is still relatively new, meaning we lack long-term perspective. Because of that, we had to widen our inclusion criteria, which creates more selection bias. Moreover, the subject of music therapy in application form is specific and these interventions are often part of a broader approach, such as an application like Calm with many options or in the presence of a therapist or professional explaining how the application works. The application modalities are not always comparable, effectiveness is sometimes indirectly demonstrated, for example, by comparing it with another care application.

The samples are generally small, almost always consisting of a few dozen or fewer individuals, severely limiting the validity of the results. The few large samples described are from online surveys, where the conditions for completion are not controlled, and the measured parameters can be considered too subjective.

Furthermore, in a broader sense, treatment provided via mobile phone may distance the patient from a potential human therapist. Even though it is not the intended goal, some institutions or authorities might consider that treatment through these applications substitutes for dialogue time with a healthcare provider. There is also a risk that patients may isolate themselves further if they find the application satisfactory or if it gives them the impression of adequate care.

To conclude, it is important to note that this review has its own limitations. Indeed, due to our broad inclusion criteria, which nevertheless led to a significant filtering among the studies found with our keywords, it is possible that a degree of subjectivity biased the selection of these studies, as well as the inherent human limitations of any research work. This subjectivity could also be reflected in our conclusions and the resulting recommendations, which should therefore be approached with caution.

Similarly, in the current state of science, it is not yet possible to establish usage recommendations for these music therapy applications; the level of evidence from the data extracted in this review is indeed largely insufficient. Nevertheless, we can discern trends from these data, which allow us to propose an example of a recommendation. This example may inspire or serve as a basis for potential future recommendations, which can only be made if further research is conducted on the subject. However, it does not represent officially validated recommendations in any way.

## 6 Example of recommendations

### 6.1 Outpatient care

#### 6.1.1 Anxiety

For mild to moderate intensity disorders, consider sessions of 15 to 20 minutes, a U-shaped sequence, a preset playlist as offered by Unwind, or one composed by the patient. These should occur at least once daily during the most anxiety-inducing moments, especially in the evening, at the workplace for work-related stress, or during the transitional period between the end of the workday and returning home, in a calm environment while resting or lying down. Prefer calming, slow, meditative music with few instruments. Schriewer et al. recommend using wind instruments and acoustic guitar.

In cases of panic attacks, consider a 15–20 minute sequence, an appropriate duration to manage the crisis, especially if the patient senses an impending attack.

#### 6.1.2 Sleep disorder

Offer a 15 to 20-minute session at bedtime, an L-shaped sequence, music listening included within a soundscape or listening to a playlist prepared in advance by or with the patient. Prioritize soothing music or, in general, calm, slow, minimally instrumentalized, gentle, and percussion-free music.

Maintain a fixed time and a calm setting, lying down, in addition to conventional hygiene and dietary rules. Consider repeating if there are awakenings during the night.

#### 6.1.3 Depressive syndrome, major depressive syndrome

Similar protocol to anxiety, at fixed times, especially during moments of the day when psychological distress is most prominent. Depending on the predominant symptoms, favor appropriate music choices. For cases with associated anxiety, prioritize relaxing music. For symptoms like inhibition, apathy, or anhedonia, opt for more stimulating music. (49) recommends energetic, fast, major-key, multi-instrumental, electronic music with piano and percussion for the stimulating valence. Adapt the music to the patient’s preferences and emphasize music associated with positive memories.

#### 6.1.4 Bipolar disorder

As a preventive measure, consider a daily 30-minute session with a balanced mix of calming and stimulating music based on the patient’s preferences, using a playlist prepared in advance.

### 6.2 Hospital use

The U-shaped sequence format could potentially be used for acute moments of anxiety, particularly as an alternative to prescribed “as-needed” medications or as a complementary approach. It may also be prescribed in addiction therapy for intense craving moments or to ease anxiety associated with bulimic episodes. Musical intervention can be offered to improve the tolerance of various medical procedures or situations, including endoscopy, lengthy examinations, MRI, or imaging procedures, for both adults and children, particularly those who are agitated or anxious. In such cases, a session just before the anxiety-inducing moment is recommended to reduce anticipatory anxiety and avoid the attentional bias of anxiety. A 10 to 20-minute U-shaped sequence or a playlist composed similarly to the anxiety protocol with music tailored to the patient’s taste, possibly selected by the patient, should be preferred. A calm and restful environment is advisable.

## 7 Conclusion

This article aims to evaluate the role of mobile music therapy applications in scientific literature and in the therapeutic arsenal of mental health care providers, while providing clear recommendations for healthcare professionals and patients.

The use of mobile applications in mental health, in general, is a relatively new and not widely known tool, and studies on this subject are quite recent, especially in the precise and narrow field of music therapy. However, many significant results emerge, especially for anxious symptoms but also for depression, other psychiatric syndromes, and symptoms where the somatic and psychological intersect, such as pain, epilepsy, or migraine.

The advantages of such therapies primarily include the absence of significant side effects, personalized content, cost-effectiveness, easy accessibility, and the universality of music. Studies have thus explored various clinical situations, in hospital settings, for both chronic and acute conditions, in elderly patients and children, and in settings such as the workplace or the home.

This article highlights the importance for a psychiatrist, during consultations, to potentially dedicate more attention to music and consider it as a therapeutic tool, enhanced by new technologies through mobile applications, much like a healthy diet or physical activity. This research opens up numerous avenues for further exploration in psychiatry, particularly the potential for music to enhance other therapies or treatments, and the possibility of developing a participatory music streaming application dedicated specifically to healthcare.

## Data availability statement

The original contributions presented in the study are included in the article/**Supplementary Material**. Further inquiries can be directed to the corresponding authors.

## Author contributions

GL: Conceptualization, Data curation, Formal analysis, Funding acquisition, Investigation, Methodology, Project administration, Resources, Software, Supervision, Validation, Visualization, Writing – original draft, Writing – review & editing. AO: Methodology, Project administration, Supervision, Visualization, Writing – review & editing, Conceptualization, Data curation, Formal analysis, Funding acquisition, Investigation, Resources, Software, Validation. AB: Conceptualization, Data curation, Formal analysis, Funding acquisition, Investigation, Methodology, Project administration, Resources, Software, Supervision, Validation, Visualization, Writing – review & editing. FF: Conceptualization, Data curation, Formal analysis, Funding acquisition, Investigation, Methodology, Project administration, Resources, Software, Supervision, Validation, Visualization, Writing – review & editing. SM: Conceptualization, Data curation, Formal analysis, Funding acquisition, Investigation, Methodology, Project administration, Resources, Software, Supervision, Validation, Visualization, Writing – review & editing.
