# Supplementary material for: Musicotherapy mobile applications: what level of evidence and potential role in psychiatric care? A systematic review
Source: Front Psychiatry. 2024 Jun 7;15:1366575. doi: 10.3389/fpsyt.2024.1366575 (PMC11190819; doi:10.3389/fpsyt.2024.1366575)
Supplement: Supplementary file 1 [file Table_1.pdf]

SUMMARY TABLE PART 1

Authors and Year	Objective	Methodology	Outcome
Carissoli et al. 2015	Evaluate the effectiveness of the "it's time to relax" mindfulness app for Android in reducing stress in adults.	Comparison with a mobile-mediated musical intervention and a control group, using the MSP, Mesure du Stress Psychologique. 32 participants, 18-day trial period.	Both intervention groups showed no significant differences. Both contributed to improved stress management, with the music group particularly effective in the subcategories of Pain and Physical Issues ($p = .026$).
Blazquez Martin et al. 2018	Evaluate m-health apps in stress management.	Study of the most important m-health apps on English and Spanish Android and iOS platforms. Categorization and rating based on impact, presence, results, languages, and functionality. 443 apps included.	The most represented category among included apps is "relaxing music," a musical intervention, accounting for 21.7% of apps (94/433). However, only 2% of apps are considered of high or medium interest.

Clarke et al. 2019	Explore interdisciplinary data describing music's ability to promote empathy and social-cultural understanding. Demonstrate that passive listening to music from an unfamiliar culture can change the listener's attitude towards that culture.	Literature review and quasi-experimental empirical study with 58 subjects, listening to Indian or West African music, measuring a change in preference for people from these cultures.	No significant change but a trend toward music influencing preference for the culture from which the music originates.
Kappert et al. 2019	Compare the stress reduction effect of a musical intervention, a spoken intervention, and both interventions together, all mediated by a mobile app.	64 participants, 3 to 10 days of intervention. Measurement of perceived stress, relaxation, stimulation, and skin conductance before and after the intervention.	Decrease in perceived stress after app use ($p < .001$), no difference between the three interventions for perceived stress or relaxation. Greater improvement in skin conductance and stimulation with music compared to other interventions. Larger decrease in perceived stress and greater increase in relaxation if the "relaxation" goal was chosen ($p < .005$). Greater increase in stimulation if the "stimulation" goal was chosen ($p = .004$)

Puzia et al. 2020	Determine parents' perception of their children's engagement with a mindfulness meditation app and the app's benefits for their children, especially in sleep.	Analysis of a cross-sectional survey of adult subscribers (N=11,108) to the Calm app. Subscribers indicating having a child under 18 (2944) were surveyed on their perceptions of their child's engagement with the app. Descriptive analysis of results.	Among the 2944 relevant participants, half (1537, 52.21%) report their child using Calm. Children primarily use Calm for sleep (76%), stress (32%), and depression or anxiety (28%). The app's musical intervention, called "music and soundscape," is the second most used (67% usage), after sleep stories (95%). 78% of parents believe the musical intervention is helpful for their children's sleep.
Hubertyet al. 2021	Evaluate the effectiveness of the Calm app for its users in terms of sleep quality and mental health.	Online survey for voluntary users. Sleep quality measured using the Pittsburgh Sleep Quality Index. Evaluation of app components, including "music/soundscapes." 9,907 participants.	Improvement in sleep quality with the musical component, especially in falling asleep ($p < .001$). Significant improvement in anxiety ($p < .001$), depression ($p < .001$), and post- traumatic stress ($p = .026$) with the musical intervention when used with the entire app.
Carling et al. 2021	Evaluate the feasibility and effectiveness of a music therapy app to aid in falling asleep and reduce anxiety in a cohort of high- level	Preliminary study involving 10 sports coaches from leagues 1 and 2, and divisions 3 and 4, using the Music Care app. Six participants tested the app daily, mainly using sequences for	Improvement in anxiety (pre- versus post-session scores: 6.0±1.0 vs. 4.3±1.5, -28%, $p < 0.0001$, effect size = 1.2 (large)) and falling asleep (62.5% (n=60) of sessions reported by participants as helpful in falling asleep).

	sports coaches.	falling asleep (64% of total).	
Taruffi 2021	Study mind wandering under the influence of music provided by an app.	Experimental study with 26 participants using an app with a playlist specifically designed to induce positive and relaxing emotions. Real- time recording of participants' thoughts, mood, and emotions by the app, using the GEMS (Geneva Emotion Music Scale)	Association between mind wandering and depression, and between mind wandering and music content (53% vs. 47%). Qualitative association between music, thought content ($p < .0001$), emotional valence ($t(224) = -1.705$, $p = .04$), and arousal ($t(224) = -2.67$, $p = .004$), the latter two constituting emotional tone.
Schlarb et al. 2021	Explore sleep apps for babies and children.	Search and classification of apps on the App Store and Google Play Store using keywords "sleep," "app," and "baby" or "children."	573 apps found. 35.9% (206) use "sleep-oriented" music, and 7.7% (44) use songs. Nineteen are installed more than five million times and primarily use music or sounds.

Hwang et al. 2021	Examine studies on the effects of mental health mobile apps on the general adult population.	Identified 1205 studies, analyzed 14.	Among the 14 studies, 1 descriptive and 13 experimental. Described apps include breathing exercises, meditation, and music therapy. They report effectiveness on stress, anxiety, depression, and well- being. Two specifically involve musical intervention: “It’s time to relax” and "mind healer."
Axelsen et al. 2022	Evaluate stress in Danish employees through crowdsourcing and test a mindfulness and music therapy app over 30 days.	Stress evaluation via a cognitive game app measuring sustained attention and working memory. Randomized controlled trial with 623 healthy volunteers in three arms: Mindfulness, music therapy, and control.	Significant improvement in sustained attention, working memory, and perceived stress in the mindfulness group ($p < .001$). 38% reduction in perceived stress in the music group. Significant correlation between effectiveness and intervention ($r(457) = 0.191$, $p < .001$). No significant difference between mindfulness and music.
Feneberg and Nater 2022	Assess the effectiveness of an immediate musical intervention on daily life stressors.	Uncontrolled pilot trial involving 10 women aged 18 to 35, exhibiting higher-than-average stress levels (Perceived Stress scale >13/40). The intervention consisted of listening to a playlist created by the subject during the stressful situation for 5	The intervention is perceived as highly satisfactory, with main effects being calming, relaxing, and distracting. The study's feasibility is good, with 46 out of 65 stressful events benefiting from all measurements. Not all results have been received yet.

		to 30 minutes. Measurement of perceived stress and saliva sampling at the moment, +15, and +30 minutes.	

SUMMARY TABLE PART 2

Authors and Year	Objectives	Methodology	Results
Schriewer et Bulaj. 2016	Study the feasibility of using music streaming services as complementary therapy for depression, anxiety, and bipolar spectrum symptoms.	Literature review on the subject.	Study of the neurological substrate of the benefits of music, the influence of musical parameters on their psychological effects. Interest in categories already present on streaming platforms. Link between EEG and EDA traces and the action of music on the psyche. Discussion on the interest of an algorithm proposing the right type of music at the right "dose." Proposal of care paths for anxiety,

			depression, and bipolar disorder and emphasis on the interest of an independent link between pharmaceutical laboratories and music streaming services.
Chai et al. 2017	Describe the neurological mechanism, theoretical basis, and potential applications of a smartphone-mediated music intervention in the management of acute and chronic pain.	Literature review on the subject.	Music delivered via smartphone can be combined with discovery algorithms to generate customized playlists to modulate pain and reduce opioid use. Music activates the reward circuit and decreases negative reinforcements related to pathological states, including chronic pain. By incorporating large databases of musical data provided by multiple users or using musical genes to create new music, music interventions can provide ever-renewed and personalized content.

Zhang et Ho 2017	Description of the development of a mobile reminiscence therapy application for dementia patients using open-source code data.	Explanation of the process, literature review on the subject.	Design in 5 days of a reminiscence music therapy application named "S3 Music Therapy," allowing patients to include personalized musical and video content for therapy.
Afra et al. 2018	Survey epileptic individuals on their preferences for mobile applications for seizure prevention, develop the rationale for digital therapy for epilepsy, create such an application, and study broader applications of these therapies for other chronic conditions.	Online questionnaire, 40 participants. For app design, literature review followed by the development of an app designed for a program of one year or more with daily interventions of 10 minutes.	Patients are widely interested in a music intervention to relieve stress (68%) in addition to seizure prevention (75%), more than any other relaxation method. Most surveyed individuals (45%) are willing to use a daily 10-minute intervention from this app for 1 year or more.

Metcalfetal. 2019	Examine the analgesic and antiepileptic effects of a specific music playlist on a mouse model. Development of the potential of an enriched environment and drug-device associations in preclinical and clinical pain and other chronic disease research.	Comparison of a musical intervention consisting of a playlist of Mozart music known for their antiepileptic effects with different analgesic drugs on groups of 5 to 8 mice with intraplantarcarrageenan injection to test pain (paw withdrawal latency measurement) and inflammation (paw thickness measurement) and corneal kindling to test epilepsy.	The musical intervention has significant analgesic ($p<.05$), anti-inflammatory ($p<.01$), and antiepileptic ($p<.05$) effects on the groups of mice.
Noel et al. 2019	Identify web, smartphone, and tablet applications usable for mental health recovery.	Survey of 63 patients with severe mental illnesses on the interest and current use of web and mobile applications and new technologies for their recovery.	Use of devices for recovery to listen to music (60%), access the internet (59%), make phone calls (59%), and send messages (54%) primarily. Interest expressed in learning to use apps for stress/anxiety management (45%), emotion management (45%), psychiatric symptom management (43%), CBT (40%), DBT (38%), and sleep (38%) to enhance their recovery.

Tak 2021	Explore the current state of tablet and mobile applications for dementia patients.	Descriptive analysis of characteristics and functional prerequisites of 83 applications found on the iTunes Store, Google, and Apple Support Community using keywords "app," "Alzheimer's," and "dementia."	Over 10% of them (n=9) specifically involve music, either for simple listening or playing music oneself. Many activities require good cognitive abilities, attention, and motor coordination, which may challenge patients. Simple activities are already beneficial for dementia patients.
Parlongue et al. 2021	Examine the impact of an application offering patient-controlled music intervention (MUSIC CARE) on episodic migraine.	20 patients with episodic migraine are included in this pilot trial. They complete an assessment of headache severity, anxiety, depression, functional impact, and medication intake pre- treatment. The intervention lasts for 3 months, with 1 to 2 music interventions (U-shaped sequence) per day and a minimum of 15 per month.	Significant reduction in migraine frequency (Mdiff = 2.8, p = .01). Significant reduction in medication intake (MDiff = 2.85, p = .02), duration of attacks (MDiff = 5.45, p = .002), anxiety (MDiff = 1.65, p = .02), and depression (MDiff = 2.45, p = .002).

Bulaj et al. 2021	Explore digital health technologies that can deliver personalized complementary care to pharmaceutical care in the treatment of cancer, depression (assessed by the ACE - Adverse Childhood Experiences - score), pain, and other chronic diseases, defining it as precision metapharmacology.	Non-systematic review of literature and news on the subject.	Numerous studies highlighting the opportunities offered by digital health technologies to promote health and a better lifestyle and manage the limitations of conventional pharmacological treatments such as non- adherence or resistance, including music therapy through mobile applications.

Hubertyet al. 2022	Investigate cancer patients, survivors, caregivers, and Calm meditation app users to develop a meditation app prototype specifically for cancer patients/survivors.	Participants recruited through social media, word of mouth, or partnerships. Daily survey during a 7-day use of the Calm app, or a single survey for existing Calm users. Three focus groups for caregivers, patients, and Calm users. Qualitative analysis of results.	27 participants. Patients explicitly request the app prototype to include a music intervention. They express that this component will be useful for both cancer patients and the general population. Overall satisfaction with Calm's music intervention for pain and anxiety expressed in testimonials by patients and caregivers.
Rodgers-Melnick et al. 2022	Evaluate the feasibility and effectiveness of a 6-session music therapy protocol on self-efficacy, quality of life, and coping abilities in individuals with sickle cell disease.	Stratified randomized clinical trial with 12 sickle cell disease adults in the control group and 12 undergoing smartphone-based music therapy for 6 sessions over 2 weeks. Daily measurement of pain, quality of life, and coping abilities.	Participants in the music intervention reported: learning new self-management skills, improved coping abilities, better pain management, enhanced self-efficacy ($p = 0.008$, $d = 1.20$), improved sleep disturbances (PROMIS scale, mean -1.49 ± 6.68, $p = 0.023$, $d = -0.99$), reduced pain interference (PROMIS -2.10 ± 4.68, $p = 0.016$, $d = -1.06$), and improved social functioning (ASCQ-Me scale 2.97 ± 6.91, $p = 0.018$, $d = 1.05$) compared to the control group.

SUMMARY TABLE PART 3

Authors and Year	Objective	Methodology	Outcome
Hansen et al. 2015	Evaluate the feasibility and effect of complementary medicines administered via mobile technology in surgical patients, including music intervention, audio relaxation technique, nature video app with and without music, on anxiety, perceived pain, and self-efficacy.	Randomized controlled trial on 105 surgical patients, with 25 for the music intervention. Evaluation of anxiety, pain, and self-efficacy at day -4, just before, just after, and day 5 post-operation.	No statistically significant results but trends towards improvement. Feasibility of the intervention in a surgical setting.
Guétin et al. 2016	Initial evaluation of an app offering music intervention (MUSIC CARE) on pain and anxiety in patients undergoing coronary angiography.	Uncontrolled observational study. 35 patients undergoing coronary angiography included, measurement of anxiety and pain before and after music intervention.	Significant reduction in anxiety ($t33 = 4.12$, $p < .0001$). No significant reduction in pain, but few patients report pain related to coronary angiography. No difference by gender.

Messika et al. 2019	Evaluate the effectiveness and feasibility of a music therapy app on acceptance and tolerance of non-invasive ventilation in acute respiratory distress care.	Prospective open-label randomized multicenter trial with 3 arms: "music intervention," "sensory deprivation," and control. The intervention consists of a 30- minute "L-shaped sequence" with the Music Care app. Follow-up for 90 days.	No significant difference for tolerance (Mean Difference 0, p=0.7). Significant difference for the post-traumatic Distress (PDI) scale (p=0.03) immediately after the intervention. Significant difference in mean and systolic blood pressure after the first NIV session (p=0.02 and 0.05 respectively). No significant difference for other parameters.
Chai et al. 2020	Evaluate the effect and feasibility of a new music application in the treatment of acute pain and anxiety in patients admitted to the emergency department.	Prospective cohort study, 81 adult patients included, listening to a 10- minute session of music from the Unwind app, with a supervised group of 38 subjects and an unsupervised group of 43 subjects. The app collects pre- and post-intervention anxiety and pain scores, and participants provide qualitative feedback on the app's acceptability. They used the PROMIS and the Pain Catastrophizing Scale (PCS).	The average pain for both groups was 6.1 on a scale of 10. A modest and significant decrease in pain is observed (mean difference of –0.81, 95% CI –0.45 to –1.16, P<.001) as well as anxiety (mean difference –0.72, 95% CI –0.33 to –1.12). Greater improvement in patients with higher initial pain, more anxiety, or more catastrophic thinking.

Guerrier et al. 2021	Evaluate the effect of a web-based music therapy app on anxiety and hypertensive events during cataract surgery.	Single-blind randomized controlled trial. 310 patients included, 1 intervention arm, 1 control arm, 155 in each arm. 20- minute music intervention before surgery.	Significantly lower incidence of hypertensive events (13.6% vs 52.9%, 95% CI, p<.001), significantly lower anxiety measured by visual scale (1.4 vs 3.1 95% CI, p=.005), and significantly lower mean sedative treatment injections during surgery (0.04 vs 0.74 95% CI, p<.001) in the music intervention arm compared to the control arm.
Yeung et al. 2021	Evaluate the evidence level and rationale of an enriched environment to reduce postoperative pain and optimize recovery in a surgical setting.	Literature review to identify and evaluate potential enriched environment techniques in perioperative pain and anxiety treatment.	The introduction of music and mobile applications, among other interventions, in the healthcare setting can improve the surgical patient's experience. Enriched environment is a simple and patient- centered means to alleviate pain and anxiety.

Awaludin et al. 2021	Creation and testing of a smartphone-mediated perioperative nursing intervention to reduce pain, anxiety, and increase early mobilization in cardiac surgery.	Research and development, controlled experimental trial, and cross-sectional study. App including therapeutic education, prayer, exercises, hypnosis, and music. Sample of 86 patients.	Significant effectiveness in reducing pain, anxiety, and increasing early mobilizations ($p<.05$). Direct effect on anxiety, indirect effect on early mobilization through anxiety.
Frasier et al. 2021	Study existing research on tablet- or smartphone-mediated therapies and their potential for managing anxiety in the pediatric oncology population during treatment.	Literature review on the subject. Recommendations proposal.	3 music therapy apps specifically described: "Six strings," "Doodle Sounds," and "Songza." Recommendation to use these interventions at all stages of care and as early as possible.
Berge et al. 2022	Describe the participatory development process of a music therapy app for dementia patients in a geropsychiatric unit and their caregivers.	Expert-led participatory design, two prototype stages, and a randomized controlled trial of the app.	Average age of participants: 82 years. 63 patient- caregiver dyads included, 13% (8) agree to use the app. High feasibility, 75% positive impact on mood, 50% positive impact on activity, and 50% describe ease of use.<br>Frequent usage for 63%.
